# Supplementary material for: Stable Pom1 clusters form a glucose-modulated concentration gradient that regulates mitotic entry
Source: eLife. 2019 May 3;8:e46003. doi: 10.7554/eLife.46003 (PMC6524964; doi:10.7554/eLife.46003)
Supplement: Transparent reporting form [file elife-46003-transrepform.docx]

***eLife’s* transparent reporting form**

We encourage authors to provide detailed information *within their submission* to facilitate the interpretation and replication of experiments. Authors can upload supporting documentation to indicate the use of appropriate reporting guidelines for health-related research (see [EQUATOR Network](http://www.equator-network.org/%20)), life science research (see the [BioSharing Information Resource](https://biosharing.org/)), or the [ARRIVE guidelines](http://www.plosbiology.org/article/info:doi/10.1371/journal.pbio.1000412) for reporting work involving animal research. Where applicable, authors should refer to any relevant reporting standards documents in this form.

If you have any questions, please consult our Journal Policies and/or contact us: [editorial@elifesciences.org](mailto:editorial@elifesciences.org).

**Sample-size estimation**

- You should state whether an appropriate sample size was computed when the study was being designed
- You should state the statistical method of sample size computation and any required assumptions
- If no explicit power analysis was used, you should describe how you decided what sample (replicate) size (number) to use

Please outline where this information can be found within the submission (e.g., sections or figure legends), or explain why this information doesn’t apply to your submission:

The number of samples, statistical test used for each experiment, and the results of that test are listed in the legend for each figure. Where possible, individual data points are plotted in addition to the arithmetic mean and standard deviation of the data set. No power analysis was used to calculate the number of samples. In general, at least 10 biological replicates (cells, particle traces) are used in each experiment. The exact number of biological and technical replicates are listed in figure legends where relevant.

**Replicates**

- You should report how often each experiment was performed
- You should include a definition of biological versus technical replication
- The data obtained should be provided and sufficient information should be provided to indicate the number of independent biological and/or technical replicates
- If you encountered any outliers, you should describe how these were handled
- Criteria for exclusion/inclusion of data should be clearly stated
- High-throughput sequence data should be uploaded before submission, with a private link for reviewers provided (these are available from both GEO and ArrayExpress)

Please outline where this information can be found within the submission (e.g., sections or figure legends), or explain why this information doesn’t apply to your submission:

The number of biological and technical replicates are detailed in the legend for appropriate figures, and individual data points are plotted in the figures where possible. For graphs where mean values measured in multiple cells are plotted, representative single-cell data are provided in figure supplements, as indicated in the text.

**Statistical reporting**

- Statistical analysis methods should be described and justified
- Raw data should be presented in figures whenever informative to do so (typically when N per group is less than 10)
- For each experiment, you should identify the statistical tests used, exact values of N, definitions of center, methods of multiple test correction, and dispersion and precision measures (e.g., mean, median, SD, SEM, confidence intervals; and, for the major substantive results, a measure of effect size (e.g., Pearson's r, Cohen's d)
- Report exact p-values wherever possible alongside the summary statistics and 95% confidence intervals. These should be reported for all key questions and not only when the p-value is less than 0.05.

Please outline where this information can be found within the submission (e.g., sections or figure legends), or explain why this information doesn’t apply to your submission:

The statistical test used for each experiment and the results of those tests are outlined in the figure legend, except where noted below. For comparisons limited to two data sets, we used a Student’s T-tests to compare differences in means. Where 3 or more data sets were compared, we used a one-way ANOVA with Tukey-Kramer test to compare the mean of each data set to that of every other data set while allowing for differences in sample size. For Figure 3C,D, we included the complete results of statistical analysis as an ANOVA table in Figure 3—figure supplement 1D. For Figure 7B,C and Figure 7—figure supplement 1A,B, we have included two supplemental tables that include the details of the linear regressions (Supplemental Table 1), and the comparisons of those linear regressions (Supplemental Table 2).

(For large datasets, or papers with a very large number of statistical tests, you may upload a single table file with tests, Ns, etc., with reference to sections in the manuscript.)

**Group allocation**

- Indicate how samples were allocated into experimental groups (in the case of clinical studies, please specify allocation to treatment method); if randomization was used, please also state if restricted randomization was applied
- Indicate if masking was used during group allocation, data collection and/or data analysis

Please outline where this information can be found within the submission (e.g., sections or figure legends), or explain why this information doesn’t apply to your submission:

Samples were allocated into experimental groups on the basis on genotype, as described in the text, figure legends, and figures. No randomization was needed or applied.

**Additional data files (“source data”)**

- We encourage you to upload relevant additional data files, such as numerical data that are represented as a graph in a figure, or as a summary table
- Where provided, these should be in the most useful format, and they can be uploaded as “Source data” files linked to a main figure or table
- Include model definition files including the full list of parameters used
- Include code used for data analysis (e.g., R, MatLab)
- Avoid stating that data files are “available upon request”

Please indicate the figures or tables for which source data files have been provided:

The genotypes of yeast strains used in this study are listed in Supplemental Table 1. These yeast strains are available for distribution upon request.
